# Association of dietary inflammatory index and vigorous physical activity on phenotypic age acceleration: a cross-sectional study with machine learning

**DOI:** 10.3389/fnut.2025.1602821

**Published:** 2025-07-28

**Authors:** Shuoqi Li, Jianming Zhou, Dandan Zhang, Qiuyu Du

**Affiliations:** ^1^School of Sports Science, Nantong University, Nantong, China; ^2^Department of Physical Education, Nanjing Xiaozhuang University, Nanjing, China; ^3^Institute of Finance and Economics, Shanghai Lida University, Shanghai, China

**Keywords:** dietary inflammatory index, vigorous intensity physical activity, phenotypic age, NHANES, machine learning

## Abstract

**Background:**

Diet and inflammation are intricately correlated to the aging process. Diet has also been hypothesized to influence aging by regulating inflammation. The phenotypic age acceleration (PhenoAgeAccel) reflects the difference between an individual’s phenotypic and chronological age; a positive value suggests accelerated aging, whereas a negative value indicates slower biological aging. Accordingly, this study investigated the independent and comprehensive influences of vigorous-intensity exercises (VPA) and dietary inflammatory index (DII) on the PhenoAgeAccel in American adults.

**Methods:**

The study enrolled 4,167 adults sourced from the National Health and Nutrition Examination Survey (NHANES) from 2007–2010 to 2015–2018. The NHANES is designed with a sophisticated, multistage probability sampling methodology and is specifically tailored to comprehensively assess the health and nutritional conditions of the non-institutionalized population. Five machine learning models were constructed to predict participants’ PhenoAgeAccel.

**Results:**

The PhenoAgeAccel of participants in Groups 3 (anti-inflammatory diet + insufficient VPA) and 4 (anti-inflammatory diet + sufficient VPA) were −2.72 (95% CI − 3.44, −1.93; *p* < 0.001), and −1.61 (95% CI − 2.65, −0.63; *p* < 0.001), respectively, when compared to the participants under 60 years old in Group 1 (pro-inflammatory diet + insufficient VPA). Conversely, a significantly increased PhenoAgeAccel was exhibited by Group 2 (pro-inflammatory diet + sufficient VPA), recording 0.81 (multivariable-adjusted *β*, 95% CI 0.13–1.75, and *p* < 0.01).

**Conclusion:**

Low levels of VPA and anti-inflammatory diet consumption were associated with reduced biological aging. Anti-inflammatory diets can also aid in counteracting the harmful effects of significant levels of VPA on biological aging.

## 1 Introduction

The aging status of an individual can be presented more appropriately by phenotypic age (PhenoAge) than chronological age, which only denotes living period and is unrelated to health status (1). PhenoAge might have arisen from molecular alterations or “hallmarks” accumulation that impede the operative and durabiliy of tissues and organs, resulting in disease and death (2, 3). Furthermore, the morbidity and mortality risks among various disease-free and healthy adults in the United States of America (USA) were reflected by phenotypic age acceleration (PhenoAgeAccel). PhenoAgeAccel reflects the difference between an individual’s phenotypic and chronological age; a positive value suggests accelerated aging, whereas a negative value indicates slower biological aging. The findings suggested that PhenoAge reflects beyond the number of comorbidities in individuals (4). In short, PhenoAge is an indicator that is closely related to an individual’s risk of adverse health outcomes and can reflect the overall extent of an individual’s aging.

Slowing the PhenoAge can hinder or stop the emergence of several age-related illnesses, possibly extending lifespan (2, 5). Human and animal studies also indicated that PhenoAge is modifiable (2). Moreover, a healthy lifestyle index, which includes a non-drinking, non-smoking, nutritious diet, physical activity (PA), and healthy body mass index (BMI), has been linked to chemistry biomarker-derived aging parameters (6, 7).

Current guidelines recommend engaging in at least 150–300 min of moderate-intensity activities (MPA), 75–150 min of vigorous-intensity exercises (VPA) weekly, or an equivalent combination to achieve health outcomes (8). VPA refers to physical activities that require higher energy consumption and significantly increase heart rate and respiratory rate, such as running, swimming, fast cycling, aerobics, tennis or squash, etc., (9). In a study (10) assessing the link between leisure-time physical activities and PhenoAgeAccel of type 2 diabetes patients, elevated conventional leisure-time physical exercise levels significantly diminished the PhenoAgeAccel. The data suggested that regular physical activities considerably reduce biological aging. Nevertheless, most studies focused on total PA or MPA. Different exercise intensity levels may have varying effects. Lu et al. (11) found that VPA exhibited the opposite effects of MPA in reducing PhenoAgeAccel. Consequently, obtaining the physiological benefits of VPA while avoiding its anti-aging side effects presents an urgent issue requiring solutions.

Advanced and intensified biological aging (12) are predominantly contributed by inflammation and age. The phenomena can result in cell dysfunction, tissue degeneration, and organ damage. Inflammation also accelerates the aging process (13), including condensed leukocyte telomere length (14), increased biological age (15), and epigenetic aging (16). Physical activity modulates inflammatory pathways and reduces oxidative stress, key processes implicated in cellular aging. While moderate exercise has anti-inflammatory effects, high-intensity or prolonged vigorous activity may initiate oxidative damage and pro-inflammatory responses in certain populations. Moreover, dietary modification provides a straightforward and manageable intervention that offers numerous health benefits (17). The dietary inflammatory index (DII) is calculated according to the inflammatory effects of dietary nutrients. The index denotes the optimal parameters for establishing the influences of food consumption practices on inflammation (18, 19) and can predict pertinent biomarkers (20).

Diet and inflammation are intricately correlated to the aging process (21). Diet has also been hypothesized to influence aging by regulating inflammation (22). Furthermore, a positive link has been demonstrated between DII and PhenoAgeAccel (23). Nonetheless, evidence regarding the influences of VPA and DII on PhenoAgeAccel is limited. Accordingly, this study investigated the independent and comprehensive influences of VPA and DII on PhenoAgeAccel in American adults. The potential correlations between the combined effects of VPA and DII on PhenoAgeAccel might provide a theoretical basis for researchers to develop reasonable and scientific dietary and exercise prescriptions.

## 2 Methods

### 2.1 Study population

**Figure 1:**
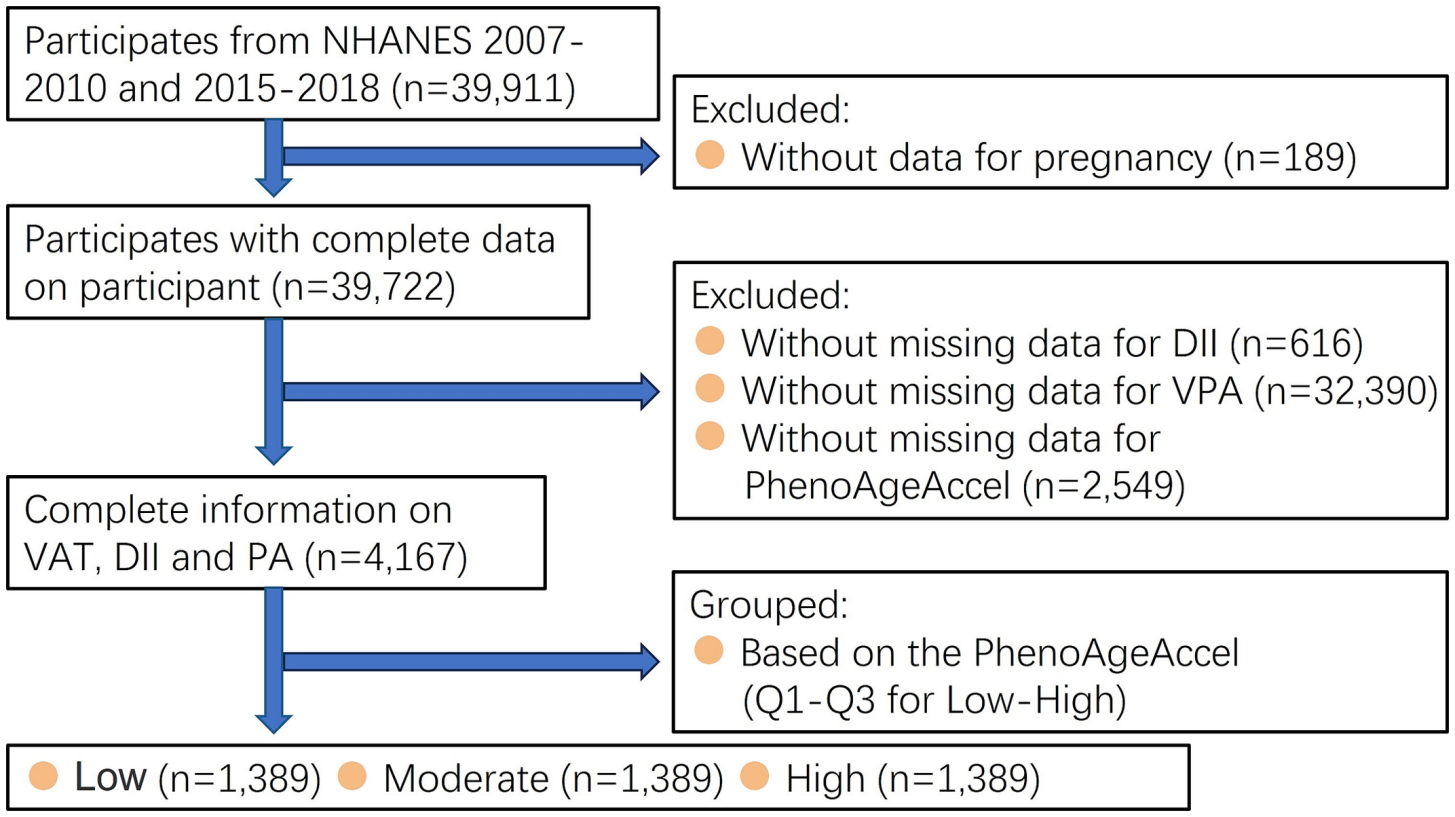
Flow chart of study participants.

Data for this study were obtained from the National Health and Nutrition Examination Survey (NHANES) database in the United States.^1^ The initiative employs a complex multistep probability sampling approach during non-institutionalized population representative selection in the USA who had provided written informed consents. The guidelines outlined in the Declaration of Helsinki was applied in the current study. A total of 39,911 adults (≥18 years old) who participated in the 2007–2010 and 2015–2018 NHANES were involved. Conversely, individuals who missed DII (*n* = 616), data about VPA (*n* = 32,390), PhenoAgeAccel (*n* = 2,549), and pregnant women (*n* = 189) were excluded. The primary analysis only included 4,167 participants (Figure 1).

### 2.2 Phenotypic age

The phenotypic ages of the participants in this study were determined based on the method described in a previous report (24). Phenotypic age is established utilizing an individual’s chronological age and nine biomarkers. The nine biomarkers for calculating PhenoAgeAccel mainly include white blood cell count, creatinine, glucose, lymphocyte percentage, mean red blood cell volume, albumin, C-reactive protein, red blood cell distribution width and alkaline phosphatase. The biomarkers in this study were selected according to the Cox proportional hazard elastic net model for death based on 10-fold cross-validation. Please refer to Supplementary Figure 3 for the detailed calculation formula of PhenoAgeAccel.

The current study established the PhenoAgeAccel of the participants based on the obtained phenotype ages. PhenoAgeAccel is the residual from a linear model following regressing phenotypic age on chronological age. Accordingly, PhenoAgeAccel denotes phenotypic age after considering chronological age. A positive value suggests accelerated aging, whereas a negative value indicates slower biological aging.

### 2.3 Vigorous intensity leisure time physical activity

According to NHANES (Questionnaire Data- Physical Activity), leisure time PA includes physical movements except work-related tasks or transportation. PA can be categorized into three classifications based on intensity: vigorous, moderate, and none. VPA encompasses recreational exercises that notably elevate heart and respiratory rates. Nonetheless, to be considered performing VPA, a minimum of ten consecutive minutes is required (9). According to the 2018 Physical Activity Guidelines for Americans, ≥75 min per week of VPA is considered sufficient, while <75 min weekly is inadequate (25).

### 2.4 DII

The comprehensive description of DII^19^ was created based on 45 potentially inflammatory dietary constituents and the corresponding representative intake (26). From the 45 components, twenty-six were selected for DII calculation. Employing the particular foods ensured a stable DII predictive power (27). Therefore, the DII score reflects the inflammatory potential of the overall diet rather than that of individual foods. Supplementary Table 1 details the dietary constituents and methods applied to calculate DII. Higher DII values denote increased dietary inflammatory potential. Consequently, the participants in this study scoring over 0 DII were defined as eating a pro-inflammatory diet. Conversely, participants documenting under 0 DII scores were consuming anti-inflammatory diet.

### 2.5 Other variables

Demographic information of the participants in the current study, including age, gender, race or ethnicity, education, marital status, household income-to-poverty ratio (PIR) (PIR: <1.3, 1.3–3.5, >3.5), were also obtained. The participants also were inquired if they had smoked at least 100 cigarettes throughout their lifetime and whether they were currently smoking. Based on the results, the participants were categorized as never, former, and current cigarette smokers.

In this study, heavy alcohol drinkers were those who consumed ≥3 drinks daily for females, ≥4 drinks per day for males, or binge drinking ≥4 drinks on some occasions for females or ≥5 drinks on some occasions for males on five or more days monthly. Meanwhile, current female moderate drinkers consumed ≥2 drinks daily, while their male counterparts had ≥3 drinks per day, or were binging on alcoholic beverages on ≥2 days a month. Participants not meeting any of the previously mentioned criteria were classified as mild, former, and never drinkers (28).

The body mass index (BMI) of the participants in the present study was established following acquiring their weights and heights at mobile examination centers, while hypertension and stroke history were extracted from databases. Meanwhile, the diabetic status of each participant was defined based on self-reported diagnosis, insulin or oral hypoglycaemic drug employments, fasting blood glucose ≥7.0 mmol/L, or glycosylated hemoglobin A1c levels ≥6.5%. The data collection process was performed according to the guidelines on the NHANES website.

### 2.6 Machine learning and definition of datasets

Split all the data into training set and test set based on 70 and 30%. Random Forest (RF), Light Gradient Boosting Machine (LGBM), Decision Tree (DT), eXtreme Gradient Boosting (XGB) and Categorical Boosting (CatBoost) were used for regression prediction of the target variables. The selected optimal model was followed by SHAP interpretability analysis.

### 2.7 Statistical analyses

Following adjusting for all covariates, a multiple linear regression analysis was conducted to determine the association between DII and VPA and PhenoAgeAccel. The interacting effects of anti-inflammatory diet consumption and VPA on PhenoAgeAccel were also analysed. In this study, the participants were divided into four groups according to their dietary inflammatory attributes and VPA. Participants with pro-inflammatory diet + insufficient VPA were in Group 1, while Group 2 included participants consuming pro-inflammatory diet + sufficient VPA. Participants with anti-inflammatory diet + insufficient VPA were in Group 3 and Group 4 participants consumed anti-inflammatory diet + sufficient VPA.

The current study conducted subgroup evaluations and restricted cubic spline (RCS) regression analysis to determine the linearity and non-linearity of the correlations between DII and PA and PhenoAgeAccel. As previously mentioned (29), the number of nodes is initially set between 3 and 5, and further determined based on the lowest Akaike information criterion value for each setting. If a non-linear relationship is found between DII, VPA, and PhenoAgeAccel, the “segmented” software package is used to determine the inflection point, relying on the results of likelihood ratio tests and bootstrap resampling methods (30). In addition, a segmented multivariate linear regression model was used to evaluate the association between DII, VPA, and PhenoAgeAccel on both sides of the breakpoint. The resultant means ± standard error (SE) indicated the participants’ continuous variables, while numbers (percentages) were employed for categorical parameters. This study fitted three statistical models, where Model 1 was not adjusted, Model 2 was adjusted for age, gender, race, and BMI, and Model 3 was further adjusted for marital status, education level, family PIR, smoking status, drinking status, hypertension, diabetes and stroke. Furthermore, subgroup analysis was conducted based on age, gender, race, BMI, marital status, education level, family PIR, smoking status, drinking status, hypertension, diabetes and stroke.

Qualified participants with missing covariate data might introduce selection bias. Consequently, the random forest interpolation approach was implemented during data interpolation to procure the missing values. Only *p*-values under 0.05 (two-sided) were considered statistically significant.

## 3 Results

### 3.1 Baseline attributes

Table 1 lists the different PhenoAgeAccel at varying levels of each basic variable. The mean (SE) age of the participants in this study was 40.19 ± 0.41 years old (data not shown). Most participants, 59.35% (*n* = 2,473), were also male (data not shown). Mexican American participants represented 15.50% (*n* = 646) of the participants, while 88.46% (*n* = 3,686) had at least a high school education (data not shown).

**Table 1:** General characteristics of participants by weight status (Mean ± SE or *N* (%)).

Characteristic	Overall (*N* = 4,167)			*p* value
	Low (*N* = 1,389)	Moderate (*N* = 1,389)	High (*N* = 1,389)	
Year (ys)	41.73 ± 0.42	40.67 ± 0.41	38.18 ± 0.39	<0.001
BMI (kg/m^2^)	25.40 ± 0.12	27.77 ± 0.15	30.51 ± 0.19	<0.001
Gender				<0.001
Male	696 (50.11%)	840 (60.48%)	937 (67.46%)	
Female	693 (49.89%)	549 (39.52%)	452 (32.54%)	
Race				<0.001
Mexican American	180 (12.96%)	242 (17.42%)	224 (16.13%)	
Other Hispanic	130 (9.36%)	163 (11.74%)	129 (9.29%)	
Non-Hispanic White	701 (50.47%)	545 (39.24%)	421 (30.31%)	
Non-Hispanic Black	152 (10.94%)	257 (18.50%)	474 (34.12%)	
Other Race	226 (16.27%)	182 (13.10%)	141 (10.15%)	
Married				<0.001
Yes	740 (53.28%)	692 (49.82%)	573 (41.25%)	
No	649 (46.72%)	697 (50.18%)	816 (58.75%)	
Education				<0.001
Less than high school graduate	131 (9.43%)	177 (12.74%)	173 (12.46%)	
High school graduate or GED	191 (13.75%)	253 (18.22%)	284 (20.45%)	
College or above	1,067 (76.82%)	959 (69.04%)	932 (67.09%)	
Smoking				<0.001
Never	905 (65.15%)	962 (69.26%)	848 (61.05%)	
Former	326 (23.47%)	257 (18.50%)	253 (18.22%)	
Current	158 (11.38%)	170 (12.24%)	288 (20.73%)	
Alcohol use				<0.001
Never	769 (55.36%)	717 (51.62%)	661 (47.59%)	
Former	472 (33.98%)	443 (31.89%)	460 (33.12%)	
Current	148 (10.66%)	229 (16.49%)	268 (19.29%)	
PIR				<0.001
<1.3	638 (45.932%)	538 (38.733%)	450 (32.397%)	
1.3–3.5	509 (36.645%)	583 (41.973%)	650 (46.796%)	
>3.5	242 (17.423%)	268 (19.294%)	289 (20.806%)	
Hypertension				0.009
Yes	1,086 (78.19%)	1,043 (75.09%)	1,017 (73.22%)	
No	303 (21.81%)	346 (24.91%)	372 (26.78%)	
Diabetes				<0.001
Yes	1,331 (95.82%)	1,282 (92.30%)	1,216 (87.55%)	
No	58 (4.18%)	107 (7.70%)	173 (12.45%)	
Stroke				0.720
Yes	1,378 (99.21%)	1,374 (98.92%)	1,375 (98.99%)	
No	11 (0.79%)	15 (1.08%)	14 (1.01%)	

Low (Q1) was defined as a phenotypic age acceleration of −13.6 to 4.3, moderate (Q2) of 4.3–13.4, and high (Q3) of 13.4–76.3. PIR, poverty impact ratio; GED, general educational development.

Notable differences were observed among three groups of the studied parameters (*p* < 0.001). The results also indicated that participants with significant PhenoAgeAccel were younger, recorded higher BMI, were males and Non-Hispanic Black, not married, less educated, had higher family income, and exhibited tendency to smoke and drink than their counterparts documenting low PhenoAgeAccel.

### 3.2 The single effect of DII or PA on PhenoAgeAccel

Table 2 shows the correlation between DII and VPA with PhenoAgeAccel. The results showed that DII was significantly positively correlated with PhenoAgeAccel. Following age, gender, race, education level, family PIR, smoking and drinking habits, and BMI adjustments, the multivariable-adjusted *β* for PhenoAgeAccel in the anti-inflammatory diet group were −2.75 (−3.39, −2.12; *p* < 0.001). Similarly, a significantly positive correlation was documented between VPA and PhenoAgeAccel. Participants in the sufficient VPA group recorded superior PhenoAgeAccel [multivariable-adjusted *β*, 1.78 (1.09, 2.48); *p* < 0.001] data to those in the insufficient VPA category.

**Table 2:** Weighted linear regression showing the relationship between DII and VPA with PhenoAgeAccel.

Group	Crude	Model 1	Model 2
DII			
≥0	0	0	0
<0	−3.50 (−4.14, −2.86) <0.001	−2.89 (−3.47, −2.30) <0.001	−2.75 (−3.39, −2.12) <0.001
VPA			
<75	0	0	0
≥75	2.83 (2.14, 3.53) <0.001	1.33 (0.68, 1.98) <0.001	1.78 (1.09, 2.48) <0.001

Model 1: Age, gender, race and BMI were adjusted; Model 2: Adjustments in Model 1 plus married, education, smorking, alcohol use, hypertension, diabetes and stroke. DII, dietary inflammatory index; VPA, vigorous physical activity.

### 3.3 DII and VPA combined association with PhenoAgeAccel among participants of varying ages

**Figure 2:**
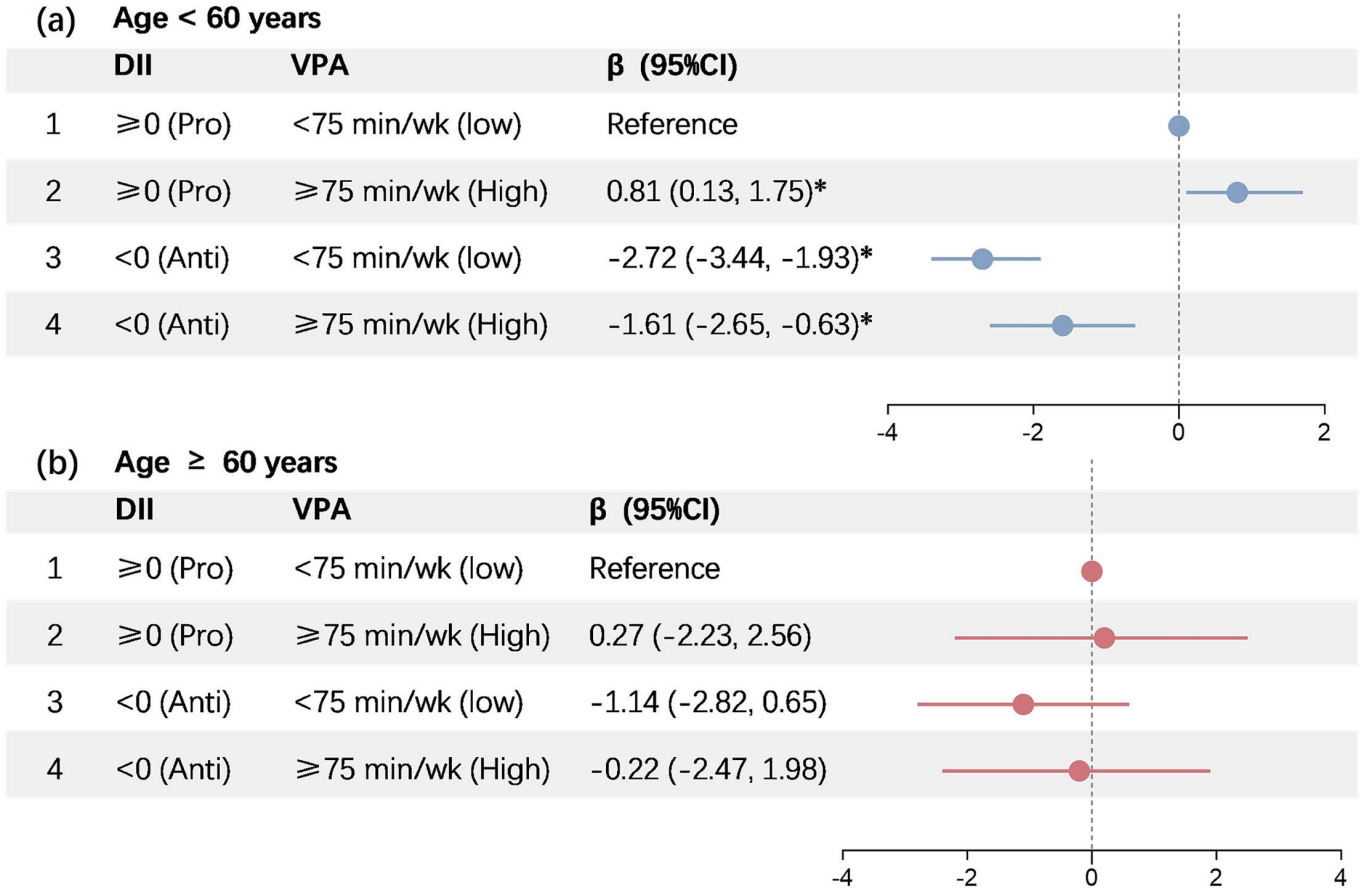
Joint association of DII and vigorous intensity PA with PhenoAgeAccel. DII, dietary inflammatory index; VPA, vigorous physical activity. *Represents significant differences compared to the baseline.

An identical set of covariates were employed in the joint analyses performed in this study. The multivariable-adjusted *β* for PhenoAgeAccel of participants in Groups 3 (anti-inflammatory diet + insufficient VPA) and 4 (anti-inflammatory diet + sufficient VPA) were −2.72 (95% CI − 3.44, −1.93; *p* < 0.001), and −1.61 (95% CI − 2.65, −0.63; *p* < 0.001), respectively, when compared to the participants under 60 years old in Group 1 (pro-inflammatory diet + insufficient VPA). Conversely, a significantly increased PhenoAgeAccel was exhibited by Group 2 (pro-inflammatory diet + sufficient VPA), recording 0.81 (multivariable-adjusted *β*, 95% CI 0.13–1.75, and *p* < 0.01) (Figure 2a). Nonetheless, no significant PhenoAgeAccel variations were observed between Groups 2 (multivariable-adjusted *β*: 27; 95% CI − 2.23, 2.56; *p* > 0.05), 3 (multivariable-adjusted *β*: −1.14; 95% CI − 2.82, 0.65; *p* > 0.05), and 4 (multivariable-adjusted *β*: −0.22; 95% CI − 2.47, 1.98; *p* > 0.05) when compared to the participants above 60 years old in Groups 1 (Figure 2b).

### 3.4 The associations between DII and VPA with PhenoAgeAccel among participants of different ages

**Figure 3:**
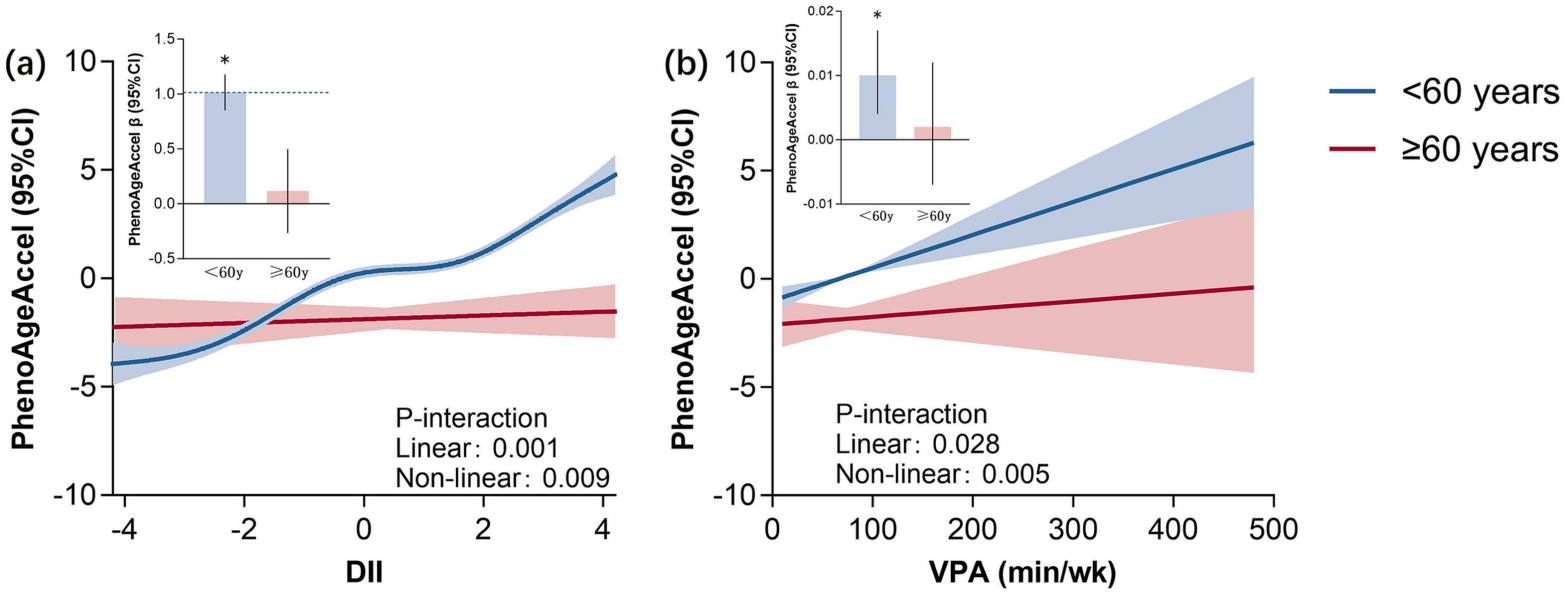
RCS plot of DII and vigorous intensity PA with PhenoAgeAccel. *Represents significant differences between two groups. DII, dietary inflammatory index; VPA, vigorous physical activity.

Figure 3 illustrates the RCS results indicating the linear and non-linear links between DII and VPA with PhenoAgeAccel. Based on the findings, varying trends were documented (*p* < 0.05). Among participants under 60 years old, the DII and VPA were significantly positively correlated with PhenoAgeAccel (*p* < 0.001). Meanwhile, no correlation was recorded between the variables in participants over 60 years old (*p* > 0.05).

### 3.5 Subgroup analyses

**Figure 4:**
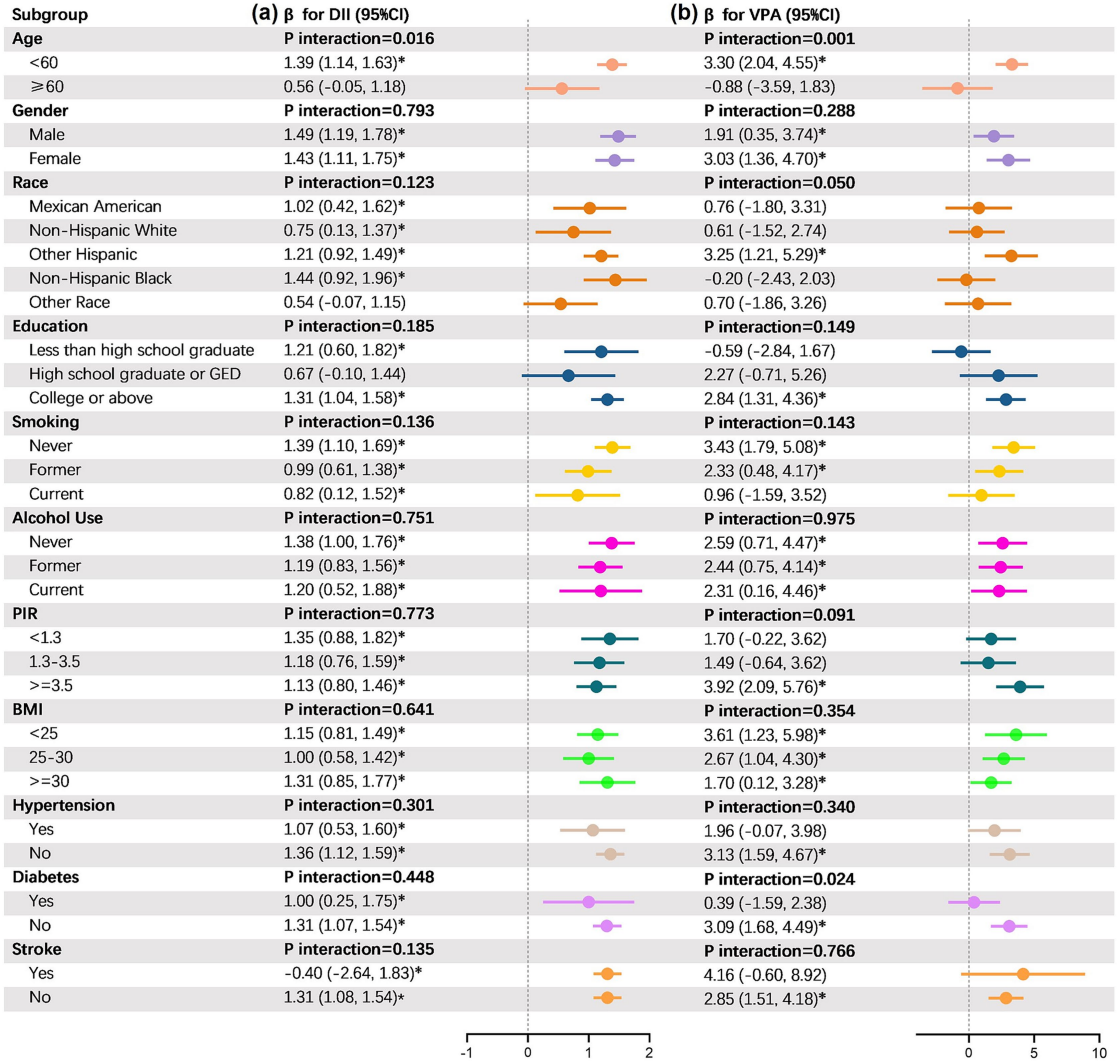
Results from subgroup analyses. DII, dietary inflammatory index; VPA, vigorous physical activity; PIR, poverty impact ratio; GED, general educational development. *Represents significant differences compared to the baseline.

The present study performed subgroup and interaction assessments based on essential covariates to further validate the robustness of the associations between DII and PA with PhenoAgeAccel across different subgroups. According to the findings demonstrated in Figure 4, the correlations remained consistent among the subgroups for the age, gender, race, education, family PIR, BMI, alcohol use, smoking, hypertension and stroke covariates. No statistically significant variations were observed across age subgroups (*p* > 0.05). The DII and VPA of participants under 60 years old were notably correlated with PhenoAgeAccel (*p* < 0.05), which was opposite to those above 60 years old.

### 3.6 Prediction performance of the validation cohort

As mentioned earlier, we used a validation cohort to verify the regression of the above RF, LGBM, DT, XGB and CatBoost machine learning models (Supplementary Figure 1). After tuning the hyperparameters, these machine learning models are trained using the entire training dataset. Their performance is subsequently evaluated using the test set. In the summary table of regression and actual information, it can be seen that the CatBoost model performs best for all parameters except the Bias parameter. In order to improve the prediction performance, we used 1,000 iterations to prevent overfitting, we eliminated concerns about covariance between predictor variables based on the regularization principle and set the following parameters for the model: maximum depth of the tree = 6, learning rate = 0.03, l2 regularization factor = 3.

The SHAP method was used to determine the importance of each predictive feature in the CatBoost machine learning model. Importance plots show the features in descending order (Supplementary Figures 2a,b). The SHA*p* values indicate the impact of each feature on the final prediction results and help to clarify the results for a particular participant. The factors that had the greatest impact on the PhenoAgeAccel among these independent variables were BMI, DII, gender, age, race, PIR, marital status, and PA. Supplementary Figures 2c,d,g show SHAP heatmaps and line plots to demonstrate the relationship between DII and PA eigenvalues and SHAP values. The higher the values of DIH and PA, the more pronounced the positive contribution to the prediction results. Meanwhile, Supplementary Figures 2e,f show the SHAP interpreted heatmaps for the RF model test set. The red bars show that the listed characteristics increased the PhenoAgeAccel in the study population, and the blue bars show that the listed characteristics decreased the PhenoAgeAccel. PA, PIR, DII and age increased the PhenoAgeAccel in our study, while on the contrary the variables of BMI, race and marital status decreased the PhenoAgeAccel (Table 3).

**Table 3:** Evaluation metric values in the validation cohort.

Model	RF	LGBM	DT	XGB	CatBoost
Bias	0.229	0.166	0.125	0.273	0.186
MAE	7.667	7.687	7.970	7.587	7.498
MAPE	2.356	2.214	2.278	2.196	2.165
RMSE	9.971	9.932	10.274	9.800	9.760
RRSE	0.893	0.889	0.920	0.877	0.874
RSE	0.797	0.791	0.847	0.770	0.764

MAE, mean absolute error; MAPE, mean absolute percentage error; RMSE, root mean squared error; RRSE, relative root squared error; RSE, residual squared error; RF, random forest; LGBM, light gradient boosting machine; DT, decision tree; XGB, eXtreme Gradient Boosting; CatBoost, Categorical Boosting.

## 4 Discussion

This study discussed the associations between DII and VPA with PhenoAgeAccel. Several key informations were also highlighted, including the significant differences in PhenoAgeAccel at varying DII levels. Specifically, anti-inflammatory diet consumption reduced the PhenoAgeAccel of the participants considerably. Similarly, low-level VPA increased PhenoAgeAccel compared to high-level VPA. The findings indicated that anti-inflammatory diets and low-level VPA have notable effects on reducing biological aging. Evaluations also revealed that although the high-level VPA lifestyle increased PhenoAgeAccel, combining anti-inflammatory diets with the VPA lifestyle could offset the side effects of high VPA on biological aging.

The results indicated age-specific effects on participants’ PhenoAgeAccel. Anti-inflammatory dietary practices and low VPA diminished the PhenoAgeAccel of participants under 60 years old, which was not observed in participants older than 60 years old. The factors that cause this result may be multi-dimensional. First of all, the problem of muscle mass loss and basal metabolic rate decline is common in the elderly (31). Degenerative changes in skeletal muscle may lead to the weakening of the promoting effect of exercise on metabolism (32). Secondly, with age, the body gradually enters a state of chronic low-grade inflammation, which is manifested by the continuous rise of circulating inflammatory markers (33). This inflammation is not caused by acute infection or injury, but is closely related to aging related immune dysregulation, mitochondrial dysfunction and cellular senescence (34). This makes the inflammation of older people more complex and more difficult to regulate (35). Finally, the decline of nutrient metabolism and absorption capacity may also lead to the decrease of bioavailability of anti-inflammatory diet (36). The results emphasized the universal role of moderate VPA and an anti-inflammatory diet in slowing biological aging. Overall, this study revealed the complex relationships between DII, VPA, and PhenoAgeAccel. Although increasing VPA is not conducive to delaying aging, the anti-inflammatory diet model can delay biological aging and offset the adverse effects of VPA.

A previous study (11) found that PA at varying intensities had different associations with aging. For instance, MPA has beneficial influences on biological aging and PhenoAgeAccel, while VPA had the opposite results. The phenomenon might be due to potentially elevating musculoskeletal complications and CVD risks, particularly in susceptible populations (37). Prolonged exercise also elevates cardiac biomarkers 31–33 and post-exercise transient myocardial dysfunction. Furthermore, endurance athletes over 35 years old exhibited enhanced myocardial late gadolinium, indicating possible fibrosis, and increased coronary artery calcium figures (38) and trial fibrillation incidences (39). The associations between the maladaptive reactions and the intensity of physical activity form a U-shaped or inverted J-shaped dose–response curve. In addition, the study of Jeremy Morris et al. showed that with the increase of exercise volume, the range of risk reduction gradually decreased, and even when the amount of activity reached the highest level, the risk may tend to be stable or slightly increased (40). The study ^40^also pointed out that long-term vigorous aerobic exercise may lead to a series of cardiac problems, including cardiomyocyte injury, myocardial fibrosis, coronary artery calcification, atrial fibrillation and aortic dilatation. PhenoAgeAccel represents an aging process intimately linked to cardiovascular homeostasis and cardiac dimensions, functions, and heart failure attack risk indicator alterations (41). Consequently, VPA may impact PhenoAgeAccel by affecting cardiovascular homeostasis. Overall, the findings suggested that VPA may contribute to adverse cardiovascular adaptation and exercise-related acute cardiac events, accelerating biological aging.

Evidence demonstrated that persistent, low-level inflammations led to cellular and tissue aging considerably, which negatively influenced biological age (42). MPA may be able to improve body inflammation by increasing the level of anti-inflammatory factors (43). Although VPA brings many health benefits, excessive exercise intensity may also cause oxidative stress, increase cortisol levels, and lead to endothelial dysfunction. These effects may lead to accelerated biological aging and increased cardiovascular risk (44). A study (44) showed that after a single HIIT (100% VO ₂ max), pro-inflammatory factors such as interleukin-6 (IL-6), TNF- *α*, and interleukin-8 increased significantly, and 2 weeks of HIIT training did not change this acute response. Nevertheless, inflammation is a vital physiological process affected by numerous variables, such as age, diet, lifestyle, immune response, genetics, and environmental conditions (45). Population data indicated that the distinct food consumption approach of individuals exerts a significant effect on balancing inflammation arising from the anti-inflammatory characteristics of the nutrients (46).

Anti-inflammatory diet can improve cardiovascular health through multi-dimensional mechanisms to neutralize the possible negative effects of VPA (47). Its core role includes inhibiting chronic inflammation, regulating lipid metabolism and improving endothelial function (46). For example, dietary patterns rich in omega-3 fatty acids, polyphenols, whole grains, vegetables and fruits can significantly reduce the levels of inflammatory markers such as C-reactive protein, IL-6 (48), and promote vasodilation by activating endothelial nitric oxide synthase, reducing the damage of oxidative stress to vascular endothelium (49). In addition, anti-inflammatory dietary patterns such as the Mediterranean diet can reduce triglycerides and low-density lipoprotein cholesterol, while increasing high-density lipoprotein cholesterol, thereby reducing the risk of atherosclerosis (50). Clinical research shows that long-term adherence to anti-inflammatory diet is associated with a 15–28% reduction in the incidence of cardiovascular disease, especially for people without metabolic syndrome (51). In a cross-sectional report involving 4,510 adults, practicing notably potential inflammatory dietary habits had elevated diseases and mortality risks correlated to chronic inflammation (52). Female participants also exhibited a positive correlation between DII and leukocyte telomere length, revealing that diet and lifestyle factors can influence telomere length through inflammation modulation (14). In conclusion, anti-inflammatory diets contribute to reduced biological aging by lowering systemic inflammation, which is a key driver of cellular senescence and tissue damage associated with aging.

Wang et al. (23) proposed a considerable effect of pro-inflammatory diets on PhenoAgeAccel. Nonetheless, the biological mechanisms of pro-inflammatory diets preventing aging remain unclear. Animal experiments indicated that pro-inflammatory diets, such as fat-rich diets, could affect gene expression, resulting in abnormal aging-related gene expressions, potentially accelerating cell aging and impeding tissue functions (53, 54). Moreover, excessive consumption of pro-inflammatory diets can disrupt gut microbiota equilibrium, trigger intestinal inflammation, and produce harmful metabolites, threatening overall health (55, 56).

Pro-inflammatory diets typically include ultra-processed foods rich in carbohydrates and fats but low in dietary fibre, and antioxidants, including vitamins C and E, carotenoids, and polyphenols. These foods are commonly associated with neuroendocrine disorders and autophagy inhibition (57, 58). Consequently, anti-inflammatory diets can counteract the effects of VPA on biological aging by improving inflammation levels.

The findings in the present study offer several notable implications, particularly in guiding healthy lifestyle behaviors. Although excessive VPA has numerous benefits, the practice might pose biological aging risks. Consuming anti-inflammatory diets of primarily green leafy and dark yellow vegetables, whole grains, fruits, coffee, and tea to counteract the adverse effects of high levels of VPA and reduce PhenoAgeAccel. Meanwhile, pro-inflammatory dietary patterns of predominantly eating red and processed meat, organ meat, refined carbohydrates, and sweetened beverages, have potential adverse effects. The findings highlighted the necessity of paying more attention to the quality of daily dietary intake.

Among the limitations of this study is that the data cannot be employed when inferring causality due to its observational approach. The food intake and VPA figures were also only baseline data, hence the effects of long-term alterations in food consumption and VPA levels during follow-ups were undetermined. In addition, the evaluation method of DII only includes 26 out of 45 foods, which limits the full discriminatory power of the index. Due to the absence of PhenoAgeAccel data, we excluded data from 2011 to 2014. Both diet and physical activity data are based on self-report, which may cause recall or reporting bias. This study also stated conclusions based on the US adults, possibly limiting their generalisability and relevance to other populations. Finally, residual or unknown confounding factors such as genetic or environmental factors could not be excluded. Future research should consider the use of longitudinal or interventional designs to explore causality in order to evaluate its impact on PhenoAgeAccel. In addition, future studies can further evaluate the effect of different PA time on PhenoAgeAccel through cohort studies or randomized controlled studies. As the current explanation of the possible mechanism of the results is only a hypothesis, it can be further verified and explored in future research.

## 5 Conclusion

Low levels of VPA and anti-inflammatory diet consumption were associated with reduced biological aging. Anti-inflammatory diets can also aid in counteracting the harmful effects of significant levels of VPA on biological aging. This study provides a potential choice of diversified strategies for the public health field. It is suggested to reduce biological aging and prevent age-related diseases through reasonable exercise and anti-inflammatory diet strategies to promote health.

## Data Availability

Publicly available datasets were analyzed in this study. This data can be found at: https://wwwn.cdc.gov/nchs/nhanes/Default.aspx.

## Footnotes

^1^ https://wwwn.cdc.gov/nchs/nhanes/Default.aspx
