# Supplementary material for: Association of dietary inflammatory index and vigorous physical activity on phenotypic age acceleration: a cross-sectional study with machine learning
Source: Front Nutr. 2025 Jul 28;12:1602821. doi: 10.3389/fnut.2025.1602821 (PMC12338044; doi:10.3389/fnut.2025.1602821)
Supplement: Supplementary file 1 [file Table_1.docx]

**Supplementary table 1.** The parameters related to dietary composition applied in the process of constructing the DII.

| Dietary composition | Overall inflammatory effect score | Global daily mean intake (units/d) | Standard deviation of the global daily intake |
| --- | --- | --- | --- |
| Alcohol (g) | -0.28 | 13.98 | 3.72 |
| Vitamin B12 (μg) | 0.11 | 5.15 | 2.70 |
| Vitamin B6 (mg) | -0.37 | 1.47 | 0.74 |
| β-Carotene (μg) | -0.58 | 3718.00 | 1720.00 |
| Caffeine (g) | -0.11 | 8.05 | 6.67 |
| Carbohydrate (g) | 0.10 | 272.20 | 40.00 |
| Cholesterol (mg) | 0.11 | 279.40 | 51.20 |
| Energy (kcal) | 0.18 | 2056.00 | 338.00 |
| Total fat (g) | 0.30 | 71.40 | 19.40 |
| Fiber (g) | -0.66 | 18.80 | 4.90 |
| Folic acid (μg) | -0.19 | 273.00 | 70.70 |
| Iron (mg) | 0.03 | 13.35 | 3.71 |
| Magnesium (mg) | -0.48 | 310.10 | 139.40 |
| MUFA (g)^c^ | -0.01 | 27.00 | 6.10 |
| Niacin (mg) | -0.25 | 25.90 | 11.77 |
| Protein (g) | 0.02 | 79.40 | 13.90 |
| PUFA (g)^d^ | -0.34 | 13.88 | 3.76 |
| Vitamin B2 (mg) | -0.07 | 1.70 | 0.79 |
| Saturated fat (g) | 0.37 | 28.60 | 8.00 |
| Selenium (μg) | -0.19 | 67.00 | 25.10 |
| Vitamin B1 (mg) | -0.10 | 1.70 | 0.66 |
| Vitamin A (RE)^b^ | -0.40 | 983.90 | 518.60 |
| Vitamin C (mg) | -0.42 | 118.20 | 43.46 |
| Vitamin D (μg) | -0.45 | 6.26 | 2.21 |
| Vitamin E (mg) | -0.42 | 8.73 | 1.49 |
| Zinc (mg) | -0.31 | 9.84 | 2.19 |

Note: a=DII of a certain dietary component = (Daily intake of the dietary component - Global daily mean intake of the dietary component) / Standard deviation of the global daily intake for the dietary component * Overall inflammatory effect score of the dietary component. The DII for each participant was obtained by summing the DII of the 26 dietary components selected in this study; b=Retinol equivalents; c=Monounsaturated fatty acids; d=Polyunsaturated fatty acids.
