# Supplementary material for: Association of dietary inflammatory index and vigorous physical activity on phenotypic age acceleration: a cross-sectional study with machine learning
Source: Front Nutr. 2025 Jul 28;12:1602821. doi: 10.3389/fnut.2025.1602821 (PMC12338044; doi:10.3389/fnut.2025.1602821)
Supplement: Supplementary file 2 [file Table_2.docx]

**Supplementary Table 2.** Threshold effect analysis table.

| AGE categorical | <60 | >=60 | VPA Total | <60 | >=60 | DII Total |
| --- | --- | --- | --- | --- | --- | --- |
| Model I |  |  | P-interaction: 0.02/ |  |  | P-interaction: 0.001 |
| Linear Trend Effect | 0.010 (0.004, 0.017) 0.0014 | 0.002 (-0.004, 0.015) 0.1979 | 0.008 (0.002, 0.014) 0.0071 | 0.998 (0.834, 1.162) <0.0001 | 0.117 (-0.265, 0.499) 0.4327 | 0.937 (0.786, 1.088) <0.0001 |
| Model II |  |  | P-interaction: 0.005 |  |  | P-interaction: 0.009 |
| Breakpoint (K) | 55 | 40 | 43 | 3.06 | -1.798 | 3.072 |
| Effect 1 in the segment < K | 0.042 (0.014, 0.070) 0.0030 | 0.194 (0.096, 0.291) 0.0001 | 0.071 (0.031, 0.112) 0.0006 | 0.842 (0.664, 1.019) <0.0001 | 1.869 (0.271, 3.468) 0.0223 | 0.788 (0.625, 0.951) <0.0001 |
| Effect 2 in the segment > K | 0.005 (-0.002, 0.013) 0.1662 | -0.022 (-0.036, -0.008) 0.0019 | 0.003 (-0.004, 0.009) 0.3897 | 5.727 (3.640, 7.814) <0.0001 | 0.154 (-0.320, 0.628) 0.5242 | 5.827 (3.815, 7.839) <0.0001 |
| Difference between Effect 2 and Effect 1 | -0.037 (-0.068, -0.005) 0.0213 | -0.216 (-0.319, -0.113) <0.0001 | -0.068 (-0.112, -0.025) 0.0021 | 4.885 (2.736, 7.035) <0.0001 | -1.715 (-3.549, 0.119) 0.0673 | 5.039 (2.971, 7.107) <0.0001 |
| Predicted Value of the Equation at the Breakpoint | 8.592 (8.070, 9.113) | 8.423 (7.332, 9.514) | 8.132 (7.660, 8.605) | 12.329 (11.652, 13.006) | 7.123 (5.750, 8.496) | 11.937 (11.317, 12.558) |
| Log-Likelihood Ratio Test | 0.021 | <0.001 | 0.002 | <0.001 | 0.061 | <0.001 |

Note: DII=Dietary inflammatory index; VPA=Vigorous physical activity. Model: Age, gender, race, BMI, married, education, smorking, alcohol use, hypertension, diabetes and stroke.
