# Supplementary figures and images for: Association of dietary inflammatory index and vigorous physical activity on phenotypic age acceleration: a cross-sectional study with machine learning
Source: Front Nutr. 2025 Jul 28;12:1602821. doi: 10.3389/fnut.2025.1602821 (PMC12338044; doi:10.3389/fnut.2025.1602821)

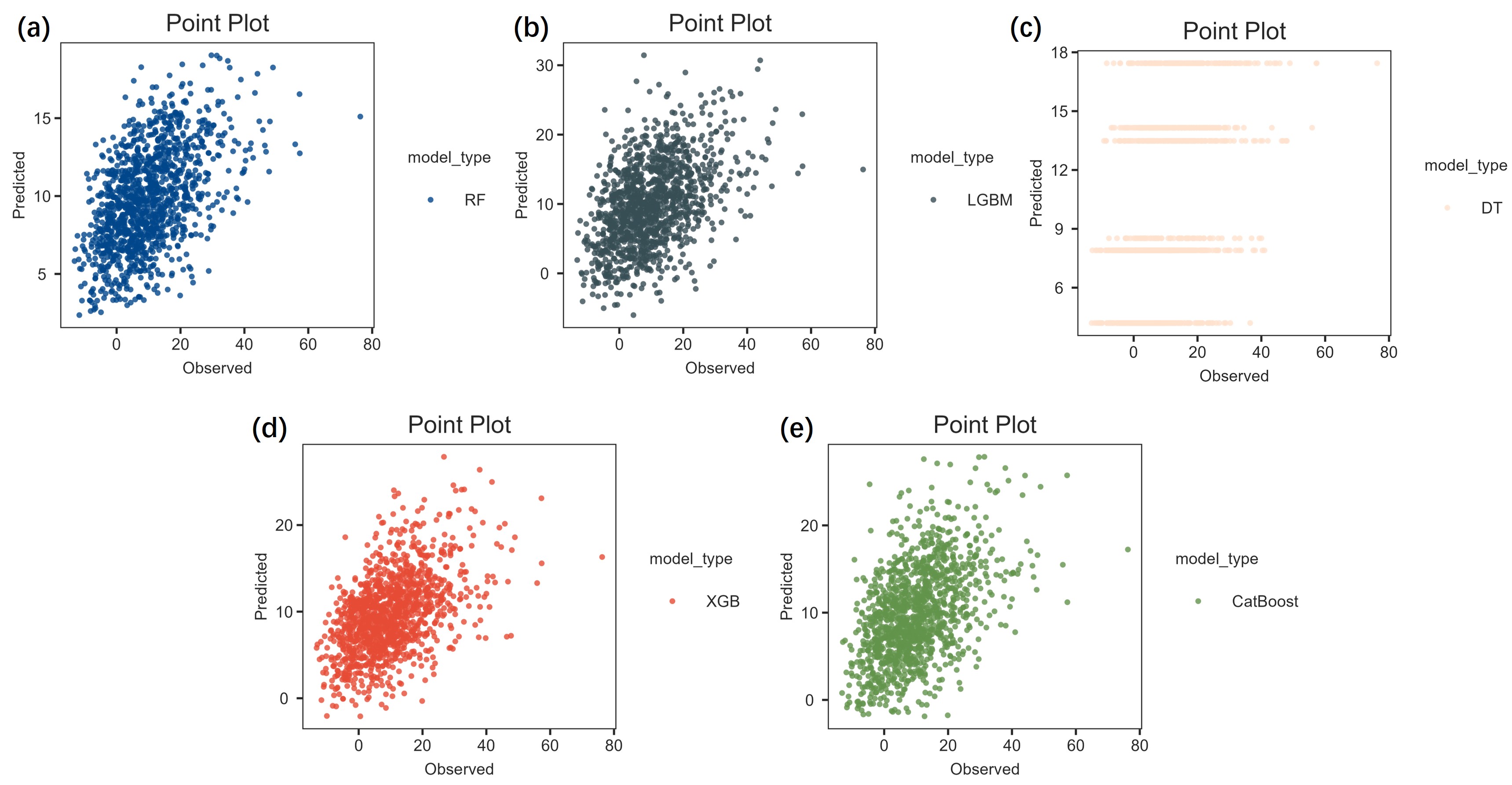

Supplement: Supplementary file 3 [file Image_1.jpeg]

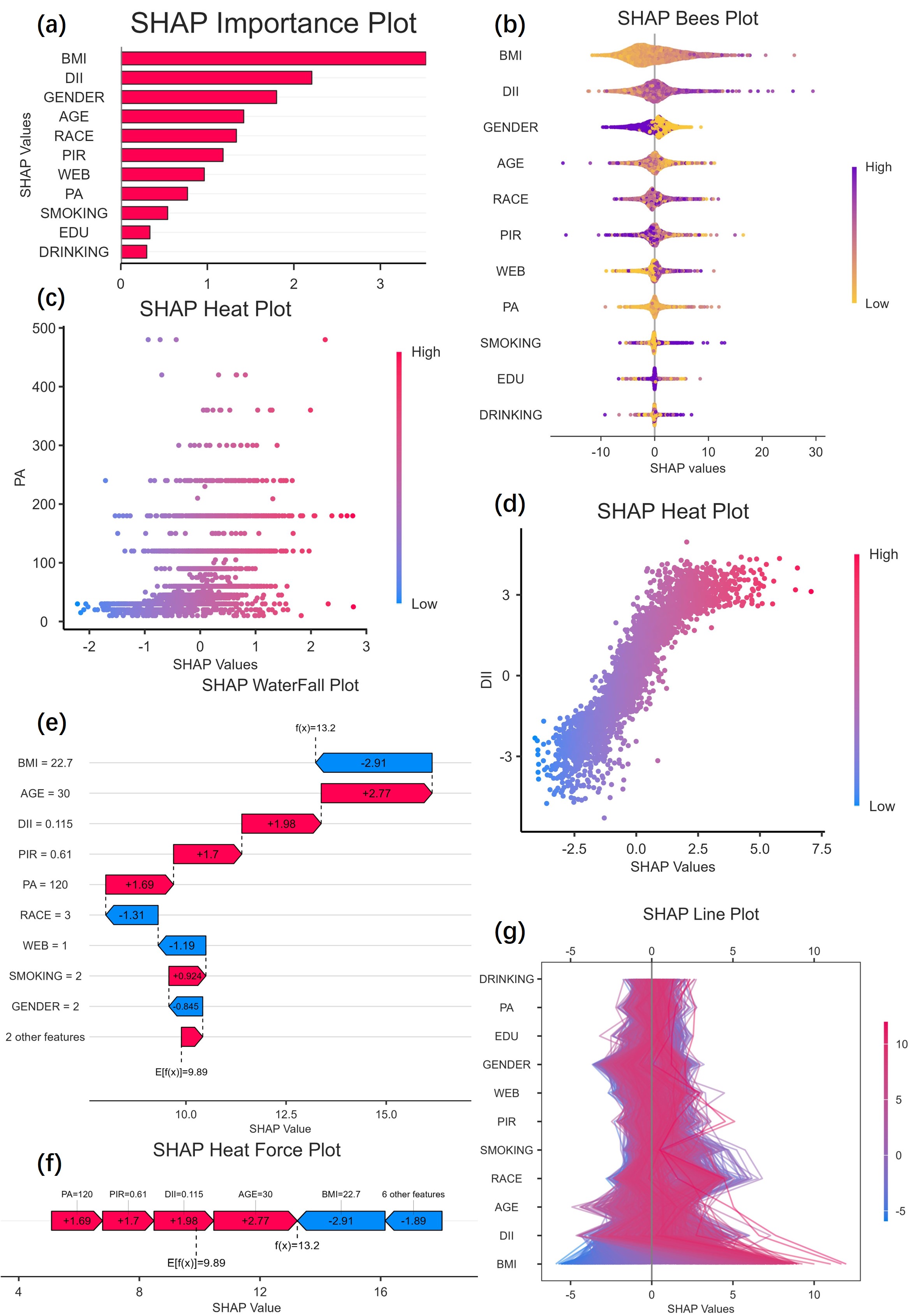

Supplement: Supplementary file 4 [file Image_2.jpeg]

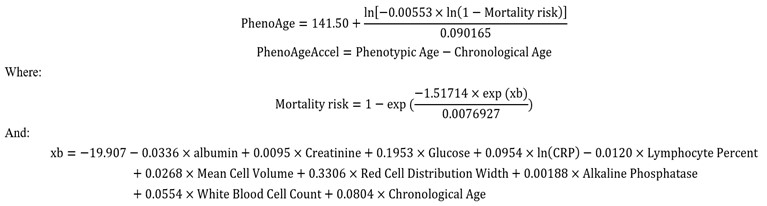

Supplement: Supplementary file 5 [file Image_3.jpeg]
